# Three pools of plasma membrane cholesterol and their relation to cholesterol homeostasis

**DOI:** 10.7554/eLife.02882

**Published:** 2014-06-11

**Authors:** Akash Das, Michael S Brown, Donald D Anderson, Joseph L Goldstein, Arun Radhakrishnan

**Affiliations:** 1 Department of Molecular Genetics, University of Texas Southwestern Medical Center, Dallas, United States; University of California, Los Angeles, United States

**Keywords:** SV-589 human fibroblasts, endoplasmic reticulum, sphingomyelin, lysosomes, cholesterol–phospholipid complexes, Perfringolysin O, *E. coli*

## Abstract

When human fibroblasts take up plasma low density lipoprotein (LDL), its cholesterol is liberated in lysosomes and eventually reaches the endoplasmic reticulum (ER) where it inhibits cholesterol synthesis by blocking activation of SREBPs. This feedback protects against cholesterol overaccumulation in the plasma membrane (PM). But how does ER know whether PM is saturated with cholesterol? In this study, we define three pools of PM cholesterol: (1) a pool accessible to bind ^125^I-PFO*, a mutant form of bacterial Perfringolysin O, which binds cholesterol in membranes; (2) a sphingomyelin(SM)-sequestered pool that binds ^125^I-PFO* only after SM is destroyed by sphingomyelinase; and (3) a residual pool that does not bind ^125^I-PFO* even after sphingomyelinase treatment. When LDL-derived cholesterol leaves lysosomes, it expands PM's PFO-accessible pool and, after a short lag, it also increases the ER's PFO-accessible regulatory pool. This regulatory mechanism allows cells to ensure optimal cholesterol levels in PM while avoiding cholesterol overaccumulation.

**DOI:** http://dx.doi.org/10.7554/eLife.02882.001

## eLife digest

Cells are enclosed by a plasma membrane that is made of lipid molecules and proteins. Almost half of the lipid molecules in the plasma membranes of animal cells (including human cells) are cholesterol molecules. Since cholesterol helps to keep the membrane stable, its level in the plasma membrane is tightly regulated.

Cholesterol is produced within animal cells, but it can also be taken up from outside the cell, primarily from low density lipoprotein (or LDL for short). When ingested LDL is broken down inside a cell, most of the cholesterol molecules are added to the plasma membrane, but some end up in the membrane of an organelle inside the cell called the endoplasmic reticulum.

The amount of cholesterol in the membrane of the endoplasmic reticulum regulates the activation of a protein called SREBP, a transcription factor that is attached to this membrane. If the level of cholesterol becomes too low, this transcription factor travels to the cell nucleus, where it switches on the genes that cause the cell to produce more cholesterol and also to take up more LDL from the environment.

When the amount of cholesterol in the membrane is high enough, the SREBP protein remains attached to the endoplasmic reticulum, which reduces the production of cholesterol and the uptake of LDL.

Here, Das et al. study the movement of cholesterol molecules between the plasma membrane and the membrane of the endoplasmic reticulum by using a toxin that binds to membranes that are rich in cholesterol. These experiments showed that the plasma membrane contains three different types or ‘pools’ of cholesterol. Das et al. found that only one of these pools is ‘labile’: it grows when there is an excess of LDL, and shrinks when cholesterol is running low in the cell. Furthermore, excess cholesterol is first added to this labile pool in the plasma membrane before it is added to the pool in the endoplasmic reticulum. This suggests that the production, uptake, and breakdown of cholesterol are all controlled by partitioning this molecule between the labile pool in the plasma membrane and the endoplasmic reticulum.

The next challenge is to determine how the three pools of cholesterol in the plasma membrane are maintained, and what regulates the distribution of cholesterol between the endoplasmic reticulum and the labile pool in the plasma membrane.

**DOI:** http://dx.doi.org/10.7554/eLife.02882.002

## Introduction

Animal cells tightly control the level of cholesterol in their plasma membranes (PMs). Control is mediated by SREBP-2, a membrane bound protein that activates transcription of genes encoding most, if not all, enzymes of cholesterol biosynthesis (Brown and Goldstein, 1997; Horton et al., 2002; Espenshade and Hughes, 2007). SREBP-2 is synthesized on endoplasmic reticulum (ER) membranes where it binds to an escort protein called Scap. When ER cholesterol is less than ∼5 mole% of total ER lipids (Radhakrishnan et al., 2008), the Scap/SREBP-2 complex enters COPII-coated vesicles that move to the Golgi, where two proteases liberate the active fragment of SREBP-2 (Sun et al., 2007). The active fragment enters the nucleus where it activates transcription of the cholesterol-synthesizing genes and also the gene for the low density lipoprotein (LDL) receptor, which supplies the cell with exogenous cholesterol (Brown and Goldstein, 1997).

When cholesterol-depleted cells are incubated with cholesterol-carrying LDL, the lipoprotein binds to its receptor, enters the cell through endocytosis in clathrin-coated vesicles and reaches lysosomes where its cholesterol is liberated (Brown and Goldstein, 1986). The lysosome-derived cholesterol is delivered to the PM and the ER membrane. When the ER cholesterol rises above a sharp threshold of 5 mole% of total ER lipids (Radhakrishnan et al., 2008), the Scap/SREBP complex binds to an ER anchor protein called Insig, and this prevents its transport to the Golgi (Goldstein et al., 2006). As a result, cholesterol synthesis and uptake from LDL are reduced. If excess cholesterol accumulates, it is removed from the PM and delivered to the ER where it is esterified by acyl-CoA:cholesterol acyltransferase (ACAT) for storage as cytoplasmic cholesteryl ester droplets (Brown and Goldstein, 1986).

The above studies expose a potential paradox. The Scap/SREBP system functions to regulate the concentration of cholesterol in the PM where the vast majority (60–90%) of a cell's total cholesterol is located (De Duve, 1971; Lange et al., 1989). Within PMs, cholesterol accounts for 40–50% of lipids (Ray et al., 1969; Lange et al., 1989; van Meer et al., 2008). Yet, the cholesterol sensor for the Scap/SREBP system is located not in the PM, but in the ER, which contains only ∼1% of a cell's total cholesterol (Lange and Steck, 1997). Within the ER, cholesterol accounts for no more than 5% of total membrane lipids. How does the small ER cholesterol pool monitor the cholesterol concentration in the large PM pool? The current paper suggests a possible resolution of this paradox.

The key to the proposed solution lies in the paths by which cholesterol moves from lysosome to the PM and to the ER. Before the current understanding of SREBP was obtained, it was suggested that LDL-derived cholesterol moves from lysosomes to the PM, and after the capacity of the PM is saturated, then secondarily to the ER (Xu and Tabas, 1991; Lange et al., 1997). Others proposed a different route whereby cholesterol moves directly from lysosomes to the ER without involving the PM (Neufeld et al., 1996; Underwood et al., 1998). Studies supporting either route rely on indirect approaches to monitor the arrival of LDL-derived cholesterol at the PM and at the ER. In all studies, arrival of cholesterol at the ER was monitored by measuring the formation of cholesteryl esters mediated by ACAT, a resident ER membrane protein (Chang et al., 1995). On the other hand, arrival of LDL-derived cholesterol at the PM was monitored using four different methods: (1) treatment of intact cells with cholesterol oxidase, a soluble cholesterol-modifying enzyme that converts cholesterol to cholestenone (Lange et al., 1997; Underwood et al., 1998); (2) treatment of intact cells with 2-hydroxypropyl-β-cyclodextrin (HPCD), a soluble acceptor that extracts cholesterol from membranes (Neufeld et al., 1996); (3) treatment of intact cells with amphotericin B, a polyene antibiotic that forms pores in cholesterol-rich membranes (Underwood et al., 1998); and (4) lipid extraction of unfractionated whole cells, followed by thin layer chromatography (Xu and Tabas, 1991). None of the aforementioned approaches directly measured the concentration of cholesterol in purified PMs.

Once LDL-derived cholesterol arrives at the PM or ER, the manner in which it is organized within each membrane also remains controversial. In particular, the distribution of cholesterol in the PM has been studied intensely. The idea that cholesterol forms complexes with PM phospholipids such as sphingomyelin (SM) in membranes has a long history (Finean, 1953; Radhakrishnan and McConnell, 2005). It is possible that these complexes exist in the PM of living cells. Under the right conditions, they could coalese to form higher-order structures, such as the proposed lipid raft domains that are enriched in cholesterol and SM and are involved in cell signaling (Brown and Rose, 1992; Simons and Ikonen, 1997). However, the size and lifetime of these lipid rafts remain hotly debated (Edidin, 2001; Munro, 2003; Gowrishankar et al., 2012). Whatever the underlying organization of cholesterol in the PM may be, reducing the SM content of PM by treatment with sphingomyelinase (SMase) causes a portion of PM cholesterol to move to the ER (Slotte and Bierman, 1988; Scheek et al., 1997; Abi-Mosleh et al., 2009).

We recently described a probe that measures an accessible pool of cholesterol in the PM (Das et al., 2013). This probe, designated ^125^I-PFO^*^, is an ^125^I-labeled, mutant version of Perfringolysin O, a bacterial protein toxin that binds to cholesterol-rich membranes (Flanagan et al., 2009; Das et al., 2013). ^125^I-PFO^*^ retains the ability to bind cholesterol, but (unlike the native version, PFO) it no longer forms pores at 4°C. (Das et al., 2013). We showed that cholesterol in PMs is not accessible to ^125^I-PFO* until the cholesterol concentration exceeds a threshold of ∼35 mole% (Das et al., 2013), which is considerably higher than the 5 mole% threshold for PFO binding to purified ER (Radhakrishnan et al., 2008; Sokolov and Radhakrishnan, 2010). Below these thresholds, PM and ER cholesterol are inaccessible to PFO.

In the current paper, we use ^125^I-PFO* binding to define three pools of cholesterol in the PM. The first pool is the ‘PFO-accessible pool’ that binds ^125^I-PFO* when the membrane is in its native cholesterol-replete state. This pool is labile, and it is depleted selectively when cells are deprived of cholesterol. The second pool binds ^125^I-PFO* only after SM in the PM is destroyed by SMase. This pool, which is not depleted by cholesterol deprivation, is referred to as the ‘SM-sequestered pool’. The remaining cholesterol exists as a third pool and does not bind ^125^I-PFO* even after SMase treatment. We name this third pool the ‘essential pool’ because depletion of this pool causes cells to round up and dissociate from the petri dish. After LDL-cholesterol is liberated in lysosomes, it is transported out of lysosomes and expands the PM's PFO-accessible pool. After a short lag, it also increases the ER's PFO-accessible regulatory pool. These data indicate how one pool of cholesterol in the PM can regulate overall cholesterol homeostasis in animal cells.

## Results

### Transport of LDL-derived cholesterol in fibroblasts: from lysosome to plasma membrane and ER

**Figure 1.**
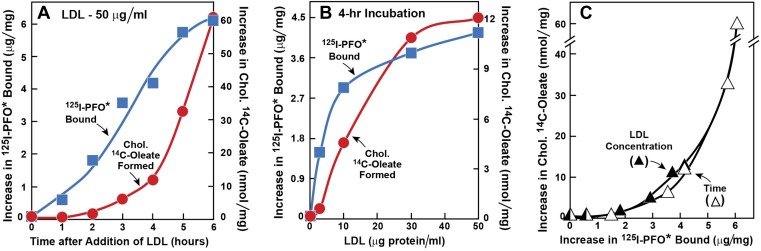
Movement of LDL-derived cholesterol from lysosomes to cell surface and to ER in human fibroblasts. On day 0, SV-589 cells were set up in medium A at 1 × 10^5^ cells per 60-mm dish. On day 2, cells were switched to lipoprotein-deficient medium C. On day 3, cells were refed with medium C containing 50 μM compactin and 50 μM sodium mevalonate and incubated for 16 hr at 37°C. On day 4, cells received 2 ml of fresh medium D containing 50 μM compactin and 50 μM mevalonate together with either 50 μg protein/ml LDL (**A**) or the indicated concentration of LDL (**B**). The cells were incubated for either the indicated time (**A**) or 4 hr (**B**) in the presence of either 0.2 mM unlabeled sodium oleate-albumin (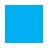) or 0.2 mM sodium [^14^C]oleate-albumin (7780 dpm/nmol) (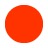). For ^125^I-PFO^*^ binding (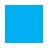), after the indicated incubation, the cells were washed five times as described in ‘Materials and methods’ and then incubated with 2 ml ice-cold buffer A containing 25 μg/ml ^125^I-PFO^*^ (11 × 10^3^ cpm/μg). After 2 hr at 4°C, the total cell surface binding of ^125^I-PFO^*^ was determined, and the amount bound after subtraction of the zero-time value (1.6 μg/mg protein) is plotted as ‘Increase in ^125^I-PFO^*^ Bound’. For measurement of cholesteryl [^14^C]oleate formation (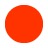), after the indicated incubation, the cells were harvested and the increase in content of cholesteryl [^14^C]oleate was determined after subtraction of the zero-time value (0.09 nmol/mg protein). All values shown are the average of duplicate incubations. (**C**) Graph showing relation between the increase in ^125^I-PFO* binding and the increase in cholesteryl [^14^C]oleate formation. Data taken from (**A**) and (**B**). **DOI:** http://dx.doi.org/10.7554/eLife.02882.003

**Figure 2.**
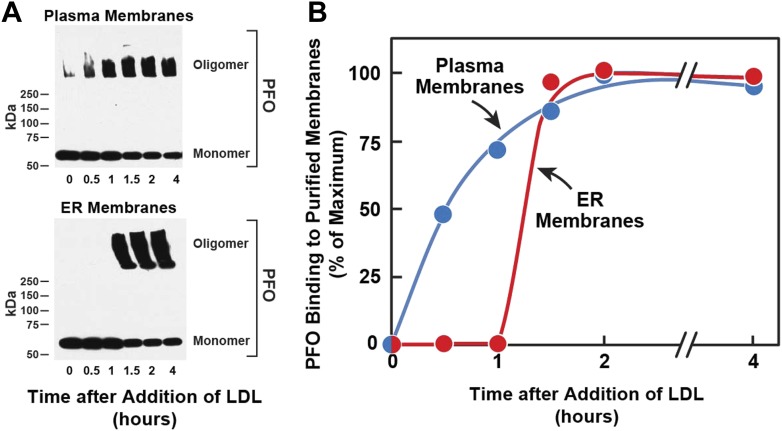
Kinetics of transport of LDL-derived cholesterol from lysosomes to PMs and ER. On day 0, SV-589 cells were set up in medium A at 1 × 10^5^ cells per 60-mm. On day 2, cells were switched to lipoprotein-deficient medium C. On day 3, cells were treated with fresh medium B containing 50 μM compactin and 50 μM sodium mevalonate and then incubated for 16 hr at 37°C. On day 4, each monolayer received 2 ml of fresh medium E containing 50 μM compactin, 50 μM mevalonate, and 50 μg protein/ml of LDL. Groups of 18 dishes were incubated at 37°C for the indicated times, after which each monolayer was washed with PBS at room temperature and then treated with 2 ml of fresh medium E containing 50 μM compactin and 50 μM mevalonate for 15 min at 37°C. Six dishes from each group were pooled for purification of PMs, and the remaining 12 dishes were pooled for purification of ER membranes, as described in ‘Materials and methods’. Purified membranes were incubated with PFO for 1 hr at 37°C as described in ‘Materials and methods’. (**A**) Oligomer formation of PFO was assessed by SDS-PAGE and immunoblotting with an antibody against the His tag. Exposure time for the films was 10 s. (**B**) Densitometric analysis of the scanned gel was performed to quantify the percentage of PFO in its oligomeric form relative to the total (oligomer + monomer) for each time point. The percentage of oligomer formed at the 2-hr time point (50% for PM; 80% for ER) was designated as 100%. **DOI:** http://dx.doi.org/10.7554/eLife.02882.004

The experiments in Figures 1 and 2 were designed to determine the time course of delivery of LDL-derived cholesterol to the ER as compared with the PM. In Figure 1, we used ^125^I-PFO^*^ binding to monitor delivery to the PM, and we used ACAT-mediated cholesterol esterification as an indirect monitor of cholesterol delivery to the ER. Human SV-589 fibroblasts were depleted of cholesterol by incubation in lipoprotein-deficient serum plus the HMG CoA reductase inhibitor compactin. We then added varying amounts of LDL together with [^14^C]oleate. After the incubation, one set of cells was harvested for measurement of cholesteryl [^14^C]oleate. The other set was chilled to 4°C and used to measure ^125^I-PFO^*^ binding. As shown in Figure 1A, when LDL was added at 50 μg protein/ml, the amount of ^125^I-PFO^*^ binding increased within 1 hr and continued to increase for 6 hr. In contrast, we detected no synthesis of cholesteryl [^14^C]oleate until after 2 hr. In Figure 1B, we fixed the incubation time at 4 hr and varied the LDL concentration. LDL at 3 μg protein/ml clearly increased the binding of ^125^I-PFO^*^ without significantly increasing cholesteryl [^14^C]oleate formation, which increased only when the LDL concentration was at least 10 μg protein/ml. Figure 1C plots cholesteryl [^14^C]oleate formation vs ^125^I-PFO^*^ binding for the data in Figures 1A,B. Irrespective of whether the incubation time or LDL concentration was varied, cholesteryl [^14^C]oleate formation did not increase until PM ^125^I-PFO^*^ binding rose above 3 μg/mg protein. These data raise the possibility that LDL-derived cholesterol must first expand a pool of cholesterol in the PM before it is delivered to the ER in sufficient amounts to undergo esterification by ACAT.

[^14^C]Oleate incorporation into cholesteryl esters and ^125^I-PFO* binding to cell surfaces are indirect measures of ER membrane cholesterol and PM cholesterol, respectively. In order to have a more direct measurement of the cholesterol content of ER and PMs, we isolated pure ER membranes and pure PMs using previously described techniques (Radhakrishnan et al., 2008; Das et al., 2013). We then estimated the accessibility of cholesterol in both the membranes by measurement of the polymerization of native PFO, which forms SDS-resistant oligomers upon binding to membrane cholesterol (Sokolov and Radhakrishnan, 2010). In the experiment of Figure 2, we first depleted cells of cholesterol and then added LDL. After varying times, one set of cells was harvested for purification of PMs, and a duplicate set was harvested for purification of ER membranes. The purified membranes were incubated with His-tagged PFO, after which the protein was solubilized with SDS-containing buffer and subjected to SDS-PAGE. SDS-resistant PFO oligomers were visualized by immunoblotting with anti-His antibody, as shown in Figure 2A. When PMs were studied, oligomers of PFO reached a half-maximal value after 30 min (top panel of Figure 2A, Figure 2B). Interaction of PFO with ER membranes was markedly different. At time periods up to 1 hr after LDL addition, PFO migrated as a monomer (bottom panel of Figure 2A, Figure 2B). At 1.5 hr, the oligomer abruptly appeared, and it did not increase further with time. These results are similar to those obtained when ER cholesterol was monitored by [^14^C]oleate incorporation into cholesteryl esters (Figure 1A).

The time-lag of 1 hr between PFO binding to PM vs ER membranes in Figure 2 does not necessarily mean that cholesterol levels in ER do not increase during this period—only that cholesterol levels have not increased past the 5 mole% threshold level for PFO binding to ER membranes. The absence of an initial lag for the PM indicates that PM cholesterol rapidly surpassed the 35 mole% threshold level for PFO binding to PM. Despite several efforts, we have been unable to measure directly the kinetics of arrival of LDL-derived cholesterol at PM and ER, owing to the difficulty in obtaining sufficient amounts of purified ER membranes that allow accurate and reproducible measurements of the mass of newly arrived cholesterol. Nonetheless, the results of Figures 1 and 2 suggest that LDL-derived cholesterol expands a pool of PM cholesterol before raising ER cholesterol by sufficient amounts to undergo esterification by ACAT or be bound by ^125^I-PFO^*^.

### Sphingomyelin sequesters a pool of cholesterol in the plasma membrane

The estimation of membrane cholesterol by the PFO oligomerization assay is non-linear and reflects the threshold nature of PFO binding to cholesterol-containing membranes. Binding of PFO to purified cell membranes does not commence until the cholesterol concentration exceeds a threshold of 5 mole% in ER membranes (Sokolov and Radhakrishnan, 2010) and 35 mole% in PMs (Das et al., 2013), respectively. One possible explanation for this higher threshold in the PM is that some of the PM cholesterol is sequestered in complexes with SM and is not accessible to PFO.

**Figure 3.**
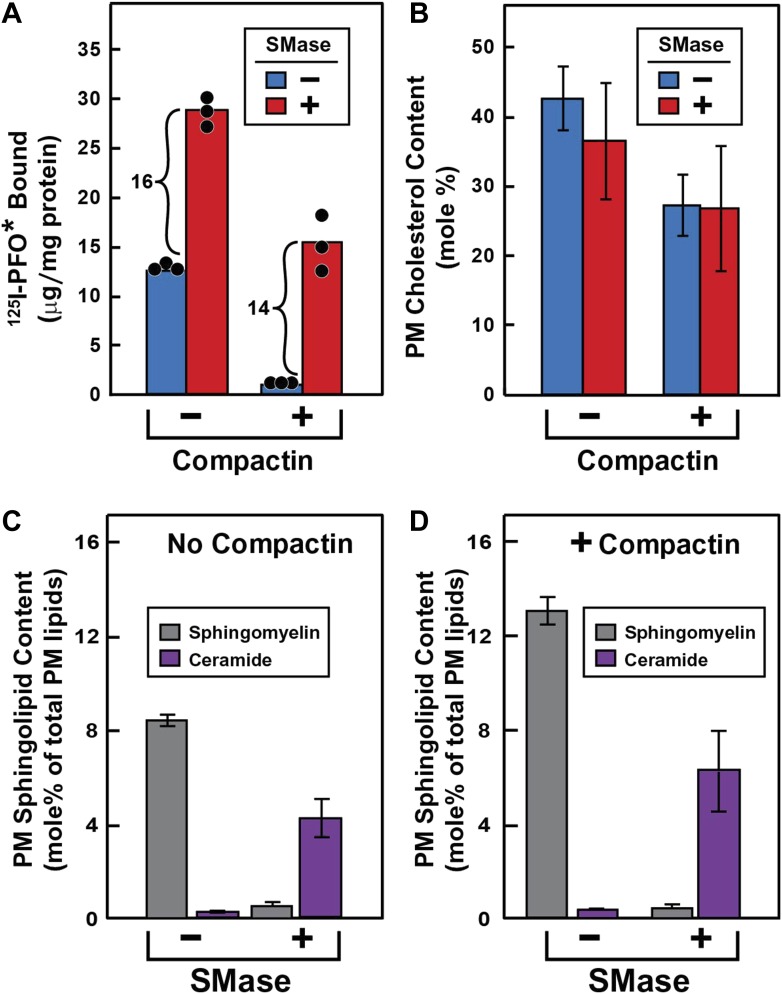
Effect of SMase treatment of human fibroblasts on amount of cell surface binding of ^125^I-PFO* (A) and PM content of SM and ceramide (B–D). On day 0, SV-589 cells were set up in medium A at 1 × 10^5^ cells per 60-mm dish (**A**–**D**). On day 2, cells were switched to lipoprotein-deficient medium C. On day 3, cells were treated with fresh medium B containing 50 μM sodium mevalonate in the presence or absence of 50 μM compactin as indicated. On day 4, each monolayer received fresh medium B containing 50 μM mevalonate in the absence or presence of 50 μM compactin and 100 milliunits/ml of SMase as indicated. (**A**) ^125^I-PFO^*^ binding. After incubation for 15 min at 37°C, the cells were washed five times as described in ‘Materials and methods’ and then incubated with 2 ml of ice-cold buffer A containing 25 μg/ml ^125^I-PFO^*^ (11 × 10^3^ cpm/μg). After 2 hr at 4°C, the total amount of cell surface binding of ^125^I-PFO^*^ was determined. Each bar represents the mean of triplicate incubations with individual values shown. (**B**–**D**) Lipid measurements. Cells were cultured under identical condition as described above. For each treatment, six 60-mm dishes were pooled together for purification of PMs by surface biotinylation as described in ‘Materials and methods’. Lipids were extracted from the membranes, and the content of cholesterol (**B**), SM (**C** and **D**), and ceramide (**C** and **D**) were measured as described in ‘Materials and methods’. The data represent the mean ± SEM obtained from three independent experiments. Each individual data point denotes the average of duplicate measurements of each pooled sample. Bracketed numbers denote the increase in ^125^I-PFO^*^ binding resulting from SMase treatment. **DOI:** http://dx.doi.org/10.7554/eLife.02882.005

To test this hypothesis (Figure 3), we treated cells without or with compactin to create cholesterol-replete or cholesterol-depleted cells. Half of the dishes from each set were treated with SMase to hydrolyze SM, after which the cells were chilled and incubated with ^125^I-PFO^*^. In cholesterol-replete cells, ^125^I-PFO* binding was abundant, and SMase treatment increased the binding by 16 μg/mg protein (Figure 3A). When the cells had been treated with compactin to deplete cholesterol, ^125^IPFO^*^ binding was reduced by 92%. Nevertheless, SMase treatment increased ^125^I-PFO* binding by 14 μg/mg protein, which was nearly the same as the increase in cholesterol-replete cells. We use the term ‘PFO-accessible’ to refer to the pool of PM cholesterol that binds to ^125^I-PFO* before SMase treatment and the term ‘SM-sequestered’ to denote the pool of PM cholesterol that is released to bind to ^125^I-PFO* after SMase treatment.

**Figure 3—figure supplement 1.**
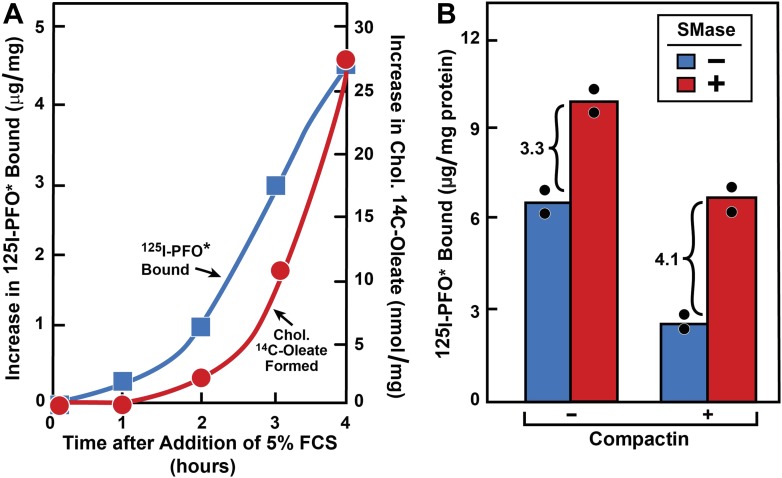
Movement of FCS-derived cholesterol and effect of SMase treatment in hamster cells. (**A**) Movement of FCS-derived cholesterol from lysosomes to cell surface and to ER in hamster cells. On day 0, CHO-7 cells were set up in lipoprotein-deficient medium G at 3 × 10^5^ cells per 60-mm dish. On day 2, cells were switched to medium G containing 50 μM compactin and 50 μM sodium mevalonate and incubated for 16 hr at 37°C. On day 3, cells received 2 ml of fresh medium H containing 50 μM compactin and 50 μM mevalonate. After incubation for the indicated time with 5% FCS (containing lipoprotein-cholesterol) in the presence of either 0.2 mM unlabeled sodium oleate-albumin (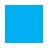) or 0.2 mM sodium [^14^C]oleate-albumin (4466 dpm/nmol) (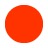), the cells were harvested for assays. For ^125^IPFO^*^ binding (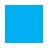), after the indicated time the cells were washed five times as described in ‘Materials and methods’ and then incubated with 2 ml ice-cold buffer A containing 25 μg/ml ^125^IPFO^*^ (45 × 10^3^ cpm/μg). After 2 hr at 4°C, the total cell surface binding of ^125^I-PFO^*^ was determined, and the amount bound after subtraction of the zero-time value (0.4 μg/mg protein) is plotted as ‘Increase in ^125^I-PFO^*^ Bound’. For measurement of cholesteryl [^14^C]oleate formation (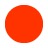), after the indicated time the cells were harvested, and the increase in content of cholesteryl [^14^C]oleate was determined after subtraction of the zero-time value (0.0 nmol/mg protein). All values shown are the average of duplicate incubations. (**B**) Effect of SMase treatment of hamster cells on amount of cell surface binding of ^125^I-PFO^*^. On day 0, CHO-K1 cells were set up in medium F at 4 × 10^5^ cells per 60-mm dish. On day 1, cells were switched to lipoprotein-deficient medium G. On day 2, cells were treated with fresh medium G containing 50 μM sodium mevalonate in the presence or absence of 10 μM compactin as indicated. On day 3, each monolayer received fresh medium G containing 50 μM mevalonate in the absence or presence of 10 μM compactin and 100 milliunits/ml of SMase as indicated. After incubation for 30 min at 37°C, the cells were washed five times as described in ‘Materials and methods’ and then incubated with 2 ml of ice-cold buffer A containing 25 μg/ml ^125^I-PFO^*^ (10.5 × 10^3^ cpm/μg). After 2 hr at 4°C, the total amount of cell surface binding of ^125^I-PFO^*^ was determined. Each bar represents the average of duplicate incubations with individual values shown. Bracketed numbers denote the increase in ^125^I-PFO^*^ binding resulting from SMase treatment. **DOI:** http://dx.doi.org/10.7554/eLife.02882.006

We also purified PMs and measured their cholesterol content directly (Figure 3B). These data show that compactin reduced PM cholesterol from 43% to 27 mole% of PM lipids (blue bars in Figure 3B), with a corresponding decrease in ^125^I-PFO* binding by 12 µg/mg protein (blue bars in Figure 3A). Using this result, we can derive an approximate relationship between ^125^I-PFO* binding and the magnitude of the PFO-accessible pool: 1 µg/mg protein of ^125^I-PFO* binding corresponds to 1.3 mole% of PM cholesterol that is PFO-accessible. We can then estimate the fraction of PM cholesterol that is in the SM-sequestered pool from the increase in ^125^I-PFO* binding (14 μg/mg) after SMase treatment to be 18 mole% of PM lipids. Considered together, these data indicate that when PM cholesterol was reduced to 27 mole% of PM lipids, little of the residual cholesterol was accessible to PFO, but 2/3 of this residual pool (18 mole%) was made accessible by SMase treatment. The remaining 1/3 of the residual pool (9 mole%) was inaccessible to PFO even after SMase treatment. The ‘Discussion’ contains a further elaboration of these cholesterol pools.

To verify that the SMase treatment depleted SM in PMs, we directly measured SM and ceramide levels in purified PMs by high-performance liquid chromatography (HPLC)-tandem mass spectrometry (MS). The distribution of the major SM species that we identified is shown in Table 1. Consistent with previous MS analysis of SM composition in human fibroblasts (Valsecchi et al., 2007), we found that more than 90% of the SM in our purified PMs was comprised of two sphingosine species, one with a palmitoyl (16:0) acyl chain and the other with a nervonoyl (24:1) acyl chain. SMase treatment reduced the levels of all SM species and produced a reciprocal increase in the corresponding ceramide species. SMase treatment of cells incubated without or with compactin reduced total SM levels in purified PM by >90% (Figure 3C) and increased total ceramide levels in these same membranes by a somewhat lesser amount (Figure 3D).

**Table 1.** Major subspecies of sphingomyelin and ceramide in sterol-repleted (−compactin) and sterol-depleted (+compactin) PMs from SV-589 cells treated without and with SMase **DOI:** http://dx.doi.org/10.7554/eLife.02882.007

Lipid	− Compactin		+ Compactin	
Species	− SMase	+ SMase	− SMase	+ SMase
mole % of total PM lipids				
Sphingomyelin				
C16:0	5.38 ± 0.19	0.32 ± 0.02	8.04 ± 0.48	0.58 ± 0.12
C18:0	0.11 ± 0.00	0.01 ± 0.00	0.16 ± 0.01	0.01 ± 0.00
C24:0	0.35 ± 0.01	0.01 ± 0.00	0.55 ± 0.03	0.02 ± 0.00
C24:1	2.59 ± 0.22	0.14 ± 0.01	4.41 ± 0.23	0.27 ± 0.05
Ceramide				
C16:0	0.04 ± 0.00	2.95 ± 0.50	0.07 ± 0.01	3.93 ± 1.22
C18:0	0.00 ± 0.00	0.11 ± 0.01	0.01 ± 0.00	0.15 ± 0.04
C24:0	0.01 ± 0.00	0.25 ± 0.04	0.01 ± 0.00	0.36 ± 0.11
C24:1	0.02 ± 0.00	1.39 ± 0.16	0.04 ± 0.00	2.14 ± 0.60

Lipids from the purified PMs isolated from the cells used in Figure 3 were extracted with 85:15 (vol/vol) ethyl acetate: isopropanol, and the contents of the four indicated acyl chain subspecies of SM and ceramide were quantified as described in ‘Materials and methods’. The data are expressed as mole % of total PM lipids and represent the mean ± SEM obtained from three independent experiments with duplicate measurements of each sample. Levels of SM and ceramide with oleoyl (18:1), arachidoyl (20:0), and behenoyl (22:0) acyl chains are not included in this table as their levels were less than 0.1% of total PM lipids.

**Figure 4.**
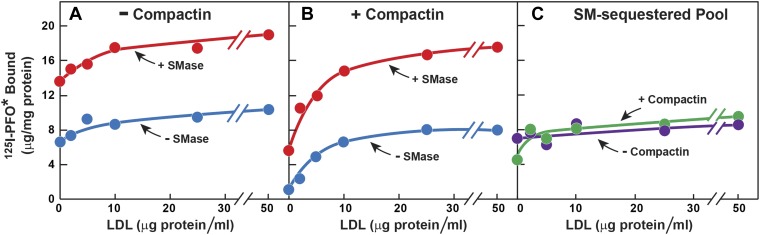
Effect of LDL on SM-sequestered pool of PM cholesterol. (**A** and **B**) On day 0, SV-589 cells were setup in medium A at 1 × 10^5^ cells per 60-mm dish. On day 2, cells were switched to lipoprotein-deficient medium C. On day 3, cells were treated with fresh medium C containing 50 μM mevalonate in the absence (**A**) or presence (**B**) of 50 μM compactin. On day 4, each monolayer received fresh medium D containing 50 μM mevalonate in the absence (**A**) or presence (**B**) of 50 μM compactin and the indicated concentration of LDL. After incubation for 5 hr at 37°C, the cells were washed twice with PBS at room temperature and treated with fresh medium D containing 50 μM mevalonate in the absence (**A**) or presence (**B**) 50 μM compactin and with or without 180 milliunits/ml SMase as indicated. After incubation for 1 hr at 37°C, the cells were washed five times as described in ‘Materials and methods’ and then incubated with 2 ml of ice-cold buffer A containing 25 μg/ml ^125^I-PFO^*^ (8 × 10^3^ cpm/μg). After 2 hr at 4°C, the total amount of cell surface binding of ^125^I-PFO^*^ was determined. Each value represents the mean of duplicate incubations. (**C**) Graph showing the SM-sequestered pool of cholesterol in the absence and presence of compactin plotted as a function of the concentration of LDL. The SM-sequestered pool was calculated by subtracting the amount of ^125^I-PFO* bound in the absence of SMase from that bound after SMase treatment. **DOI:** http://dx.doi.org/10.7554/eLife.02882.008

The striking aspect of Figure 3A is that the SM-sequestered pool of cholesterol was nearly identical whether or not the total membrane cholesterol had been depleted with compactin. To determine whether this constancy is maintained over a broad range of PM cholesterol concentrations, we incubated cells with or without compactin and then exposed them to varying levels of LDL to increase PM cholesterol prior to measurement of ^125^I-PFO^*^ binding (Figure 4). In the absence of compactin treatment, ^125^I-PFO^*^ binding was relatively high in the absence of LDL, and the binding rose only slightly when increasing amounts of LDL were added (Figure 4A). SMase treatment increased ^125^I-PFO^*^ binding at all concentrations of LDL, and the shape of the curve was parallel to the curve without SMase. When the cells were treated with compactin in the absence of LDL, ^125^I-PFO binding was very low (Figure 4B), and it rose to a saturating value when increasing amounts of LDL were added. SMase treatment increased ^125^I-PFO^*^ binding at all LDL concentrations, and the saturation curve paralleled the curve in the absence of SMase. Figure 4C plots the SM-sequestered pool as a function of LDL concentration. This pool is defined as the difference between ^125^I-PFO^*^ binding in the presence and absence of SMase. Whether or not the cells had been treated with compactin, the SM-sequestered pool remained remarkably constant at all LDL concentrations. The only deviation occurred in the compactin-treated cells in the absence of LDL, where PM cholesterol levels were very low. In this extreme example of cholesterol depletion, the SM-sequestered pool declined slightly. Using the conversion factor defined by Figure 3 and the ^125^I-PFO^*^ binding value of the increase in the PFO-accessible pool (9 µg/mg, Figure 4C), we estimate the SM-sequestered cholesterol pool in this experiment to be 12 mole% of PM lipids, which is somewhat lower than the 18% value calculated for Figure 3.

### Release of cholesterol from sphingomyelin-sequestered pool does not require vesicular transport

**Figure 5.**
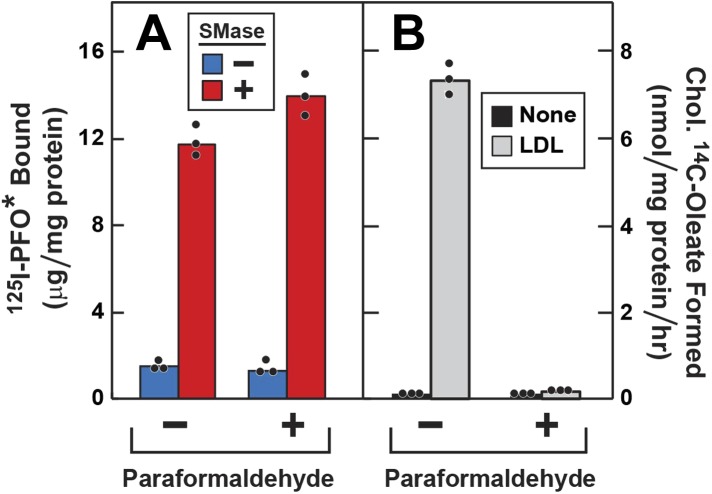
Effect of paraformaldehyde on ^125^I-PFO* binding to human fibroblasts after treatment with SMase. On day 0, SV-589 cells were setup in medium A at 1 × 10^5^ cells per 60-mm dish. On day 2, cells were switched to lipoprotein-deficient medium C. On day 3, cells were treated with fresh medium C containing 50 μM compactin and 50 μM mevalonate and incubated for 16 hr at 37°C. On day 4, each monolayer was washed with ice-cold PBS at 4°C and treated with 2 ml ice-cold PBS in the presence or absence of 1% (vol/vol) paraformaldehyde as indicated. After incubation at 4°C for 45 min, the paraformaldehyde-containing medium was removed, and each monolayer was washed four times with buffer A at room temperature. (**A**) ^125^I-PFO^*^ binding. After treatment with paraformaldehyde and washing, cells were incubated with 2 ml of fresh medium D containing 50 μM mevalonate and 50 μM compactin in the presence or absence of 100 milliunits/ml SMase as indicated. After incubation for 30 min at 37°C, the cells were washed five times as described in ‘Materials and methods’ and then incubated with 2 ml of ice-cold buffer A containing 25 μg/ml ^125^I-PFO^*^ (64 × 10^3^ cpm/μg). After 2 hr at 4°C, the total amount of cell surface binding of ^125^I-PFO^*^ was determined. (**B**) Cholesterol esterification. After treatment with paraformaldehyde and washing, each monolayer received 2 ml of fresh medium D containing 50 μM mevalonate and 50 μM compactin in the presence or absence of 50 μg protein/ml LDL. After incubation for 4 hr, 0.2 mM sodium [^14^C]oleate-albumin (7733 dpm/nmol) was added to the cell and incubated for additional 2 hr. After the desired incubation, the cells were harvested and the content of cholesteryl [^14^C]oleate was determined. (**A** and **B**) Each bar represents the mean of triplicate incubations with the individual values shown. Paraformaldehyde treatment did not significantly affect the cellular uptake of [^14^C]oleate as indicated by parallel measurements of the incorporation of [^14^C]oleate into [^14^C]triglycerides. In the absence of paraformaldehyde, the amount of [^14^C]triglycerides formed (nmol/mg protein per hr) was 51 (no addition) and 43 (+LDL); in the presence of paraformaldehyde, the values were 57 (no addition), and 32 (+LDL). These triglyceride values represent the mean of triplicate incubations. In a separate experiment, the aforementioned mean triglyceride values were 74, 46, 57, and 66 nmol/mg per hr, respectively. **DOI:** http://dx.doi.org/10.7554/eLife.02882.009

To determine whether the increase in ^125^I-PFO^*^ binding after SMase treatment requires vesicular movement, we pretreated the cells with paraformaldehyde to fix the PM prior to SMase treatment (Brown et al., 1976). First, the cells were depleted of cholesterol by incubation with compactin, and then they were treated with SMase with or without prior paraformaldehyde treatment. In the absence of paraformaldehyde, SMase caused a large increase in ^125^I-PFO^*^ binding. From the increase in ^125^I-PFO^*^ binding in Figure 5A, we estimate the SM-sequestered cholesterol pool to be 13 mole% of PM lipids. This increase was undiminished after paraformaldehyde treatment (red bars in Figure 5A). To show that paraformaldehyde was active, we incubated the cells with LDL and measured the incorporation of [^14^C]oleate into cholesteryl [^14^C]oleate (Figure 5B). Stimulation of esterification requires the receptor-mediated endocytosis of LDL (Brown and Goldstein, 1986). In the absence of paraformaldehyde, LDL caused a major increase in cholesteryl ester synthesis, and this was totally blocked by paraformaldehyde. Paraformaldehyde did not inhibit the incorporation of [^14^C]oleate into [^14^C]triglycerides (see legend to Figure 5).

### Cholesterol released from sphingomyelin-sequestered pool is free to translocate to ER

**Figure 6.**
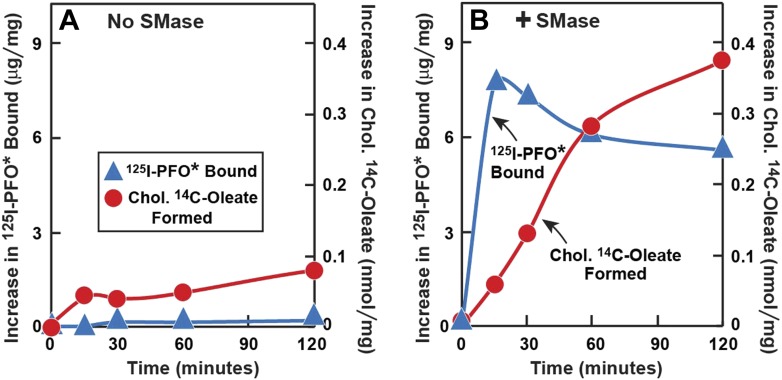
Time course of ^125^I-PFO* binding and cholesterol esterification in human fibroblasts after treatment with SMase. On day 0, SV-589 cells were setup in medium A at 1 × 10^5^ cells per 60-mm dish. On day 2, cells were switched to lipoprotein-deficient medium C. On day 3, cells were treated with fresh medium C containing 50 μM compactin and 50 μM mevalonate and incubated for 16 hr at 37°C. On day 4, each dish received fresh medium D containing 50 μM compactin and 50 μM mevalonate. Half of the dishes received 143 milliunits/ml of SMase in the absence (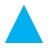) or presence (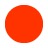) of 0.2 mM sodium [^14^C]oleate-albumin (7931 dpm/nmol) for the indicated time. For ^125^I-PFO^*^ binding (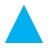) , after the indicated incubation the cells were washed five times as described in ‘Materials and methods’ and then incubated with 2 ml ice-cold buffer A containing 25 μg/ml ^125^I-PFO^*^ (10 × 10^3^ cpm/μg). After 2 hr at 4°C, the total cell surface binding of ^125^I-PFO^*^ was determined, and the amount bound after subtraction of the zero-time value (1 µg/mg protein) is plotted as ‘Increase in ^125^I-PFO^*^ Bound’. For measurement of cholesteryl [^14^C]oleate formation (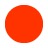), the cells were harvested and the content of cholesteryl [^14^C]oleate was determined. Each value represents the average of duplicate incubations. **DOI:** http://dx.doi.org/10.7554/eLife.02882.010

SMase treatment has been reported to cause cholesterol to translocate from the PM to the ER where it is esterified (Slotte and Bierman, 1988; Scheek et al., 1997; Abi-Mosleh et al., 2009). This predicted cholesterol loss from the PM conflicts with the increase in PFO-accessible cholesterol that is detected with ^125^I-PFO^*^. To resolve this issue, we performed a time course experiment (Figure 6). Human fibroblasts were treated with compactin to deplete cholesterol and then incubated with [^14^C]oleate in the absence or presence of SMase for varying times. After the incubation, one set of cells was harvested for measurement of cholesteryl [^14^C]oleate, and the other was chilled and incubated with ^125^I-PFO^*^. When incubated in the absence of SMase, the cells incorporated a very small amount of [^14^C]oleate into cholesteryl [^14^C]oleate, and ^125^I-PFO^*^ binding was also low (Figure 6A). In the presence of SMase (Figure 6B), ^125^I-PFO^*^ binding rose rapidly, reaching a peak at 15 min, after which it fell by about 20%. In contrast, the content of cholesteryl [^14^C]oleate was low at 15 min, and it rose progressively throughout the rest of the incubation. These data indicate that SM depletion first releases cholesterol into a PFO-accessible pool, and then the excess cholesterol is transported to the ER. The absolute amount of cholesteryl [^14^C]oleate formed at 2 hr (0.38 nmol/mg) was much less than we generally observed when LDL is added (for example, 7 nmol/mg per hr in Figure 5B). This indicates that only a small amount of cholesterol moves to the ER after SMase treatment. From the increase in ^125^I-PFO^*^ binding in Figure 6B, we estimate the SM-sequestered cholesterol pool to be 10 mole% of PM lipids in this experiment.

**Figure 7.**
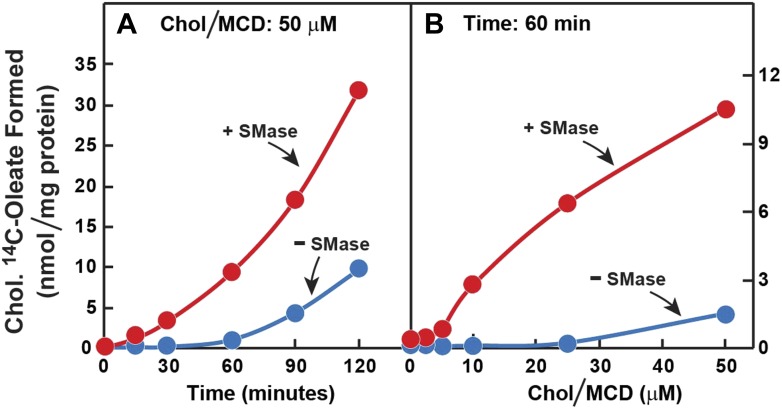
Prior incubation of human fibroblasts with SMase stimulates transport of cholesterol from PM to ER. On day 0, SV-589 cells were set up in medium A at 1 × 10^5^ cells per 60-mm dish. On day 2, cells were switched to lipoprotein-deficient medium C. On day 3, cells were treated with fresh medium C containing 50 μM compactin and 50 μM mevalonate and then incubated for 16 hr at 37°C. On day 4, each monolayer received 2 ml of fresh medium C containing 50 μM mevalonate and 50 μM compactin in the absence or presence of 100 milliunits/ml of SMase as indicated. After incubation with SMase for 15 min at 37°C, the cells in (**A**) received a direct addition of 50 cholesterol/MCD together with 0.2 mM sodium [^14^C]oleate-albumin (8572 dpm/nmol) and were then incubated for the indicated time, and the cells in (**B**) received a direct addition of the indicated concentrations of cholesterol/MCD together with 0.2 mM sodium [^14^C]oleate-albumin (6948dpm/nmol) and were then incubated for 1 hr. After the indicated incubations, the cells were harvested and the content of cholesteryl [^14^C]oleate was determined. Each value represents the average of duplicate incubations. **DOI:** http://dx.doi.org/10.7554/eLife.02882.011

The preceding data suggest that SMase treatment increases the transfer of LDL-derived cholesterol from the PM to the ER. To study this transfer process more directly, we first depleted cells of cholesterol by treatment with compactin. Next, we treated the cells with SMase and then added cholesterol directly to the PM by delivering it in a complex with methyl-β-cyclodextrin (MCD). Transfer to ER was assessed by measurement of [^14^C]oleate incorporation into cholesteryl [^14^C]oleate. After SMase treatment, the cholesterol-stimulated esterification reaction was markedly increased whether examined over time (Figure 7A) or as a function of the concentration of cholesterol/MCD (Figure 7B).

### Sphingomyelinase treatment does not release all cholesterol from plasma membrane, leaving a residual ‘essential’ pool

**Figure 8.**
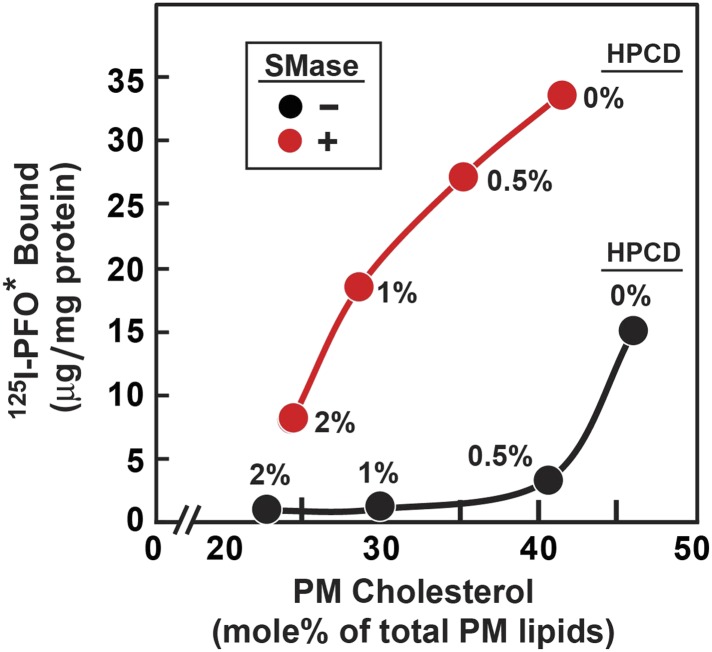
Reduced threshold for ^125^I-PFO* binding after treatment of human fibroblasts with SMase. On day 0, SV-589 cells were set up in medium A at 1 × 10^5^ cells per 60-mm dish. On day 3, cells were refed with medium B. On day 4, cells were treated for 1 hr at 37°C in medium B containing 50 µM mevalonate, 50 µM compactin, and 0–2% of HPCD as indicated for each point. The cells were then washed twice with PBS at room temperature, after which each monolayer received fresh medium B containing 50 µM compactin and 50 µM mevalonate in the absence or presence of 100 miliunits/ml of SMase. After incubation for 30 min at 37°C, one set of dishes was used for ^125^I-PFO^*^ binding and a parallel set was used for PMs purification. For ^125^I-PFO* binding, cells were washed five times as described in ‘Materials and methods’ and then incubated with 2 ml ice-cold buffer A containing 25 µg/ml ^125^I-PFO^*^ (10 × 10^3^ cpm/µg) for 2 hr at 4°C. The total amount of cell surface ^125^I-PFO^*^ binding was determined. Each value represents the average of duplicate incubations. For PM purification, six 60-mm dishes for each HPCD treatment were pooled together, and the PMs were purified as described in ‘Materials and methods’. Lipids were extracted from the PM samples, and the content of unesterified cholesterol, phospholipids, and ceramide was measured. Each value represents the average of duplicate measurements of each pooled sample. The graph shows the amount of cell surface ^125^I-PFO^*^ binding plotted as a function of the cholesterol content of the PM. **DOI:** http://dx.doi.org/10.7554/eLife.02882.012

Figure 8 shows an experiment in which we compared the threshold for ^125^I-PFO^*^ binding to cells treated without and with SMase. For this purpose, cholesterol was removed from cells by exposure to concentrations of 2-hydroxypropyl-β-cyclodextrin (HPCD) ranging from 0% to 2%. Half of the dishes were then treated with SMase, after which ^125^I-PFO^*^ binding was measured. A parallel set of dishes was harvested for measurement of the cholesterol concentration in isolated PMs. In the absence of SMase treatment, ^125^I-PFO^*^ binding decreased substantially when PM cholesterol was reduced below 45 mole% of total PM lipids (black curve). In the absence of cholesterol depletion (0% HPCD), SMase treatment increased ^125^I-PFO^*^ binding by 18 µg/mg protein. Using the conversion factor defined by Figure 3 and the ^125^I-PFO^*^ binding value of the increase in the PFO-accessible pool (18 µg/mg), we estimate the SM-sequestered cholesterol pool to be 23 mole% of PM lipids. This SM-sequestered pool declined gradually as the concentration of HPCD increased (red curve). When PM cholesterol fell to 25 mole% of total PM lipids, the SM-sequestered pool had declined by 80%. The residual cholesterol was in a pool that was not accessible to ^125^I-PFO^*^ even after SMase treatment. We call this pool the ‘essential’ pool (‘Discussion’). This ‘essential pool’ may be related to the fraction of PM cholesterol in fibroblasts that fails to be modified by cholesterol oxidase after prior treatment with SMase (Pörn and Slotte, 1995).

## Discussion

The current studies help to resolve a conundrum in cellular cholesterol regulation that has existed ever since the discovery of SREBPs 20 years ago. How does a transcription factor residing in the cholesterol-poor ER membrane regulate the cholesterol content of the cholesterol-rich plasma membrane (PM)? Here, we show how the controlled distribution of LDL-derived cholesterol to PM and ER accomplishes this regulatory task. As long as the newly expanding LDL-derived and PFO-accessible PM cholesterol pool remains low, little cholesterol moves to the ER, SREBPs are transported to the Golgi for processing, and the cell acquires cholesterol from endogenous synthesis and uptake of LDL. As this PM pool expands, more cholesterol moves to the ER. Once this ER cholesterol surpasses a 5 mole% threshold, it blocks SREBP transport, reducing the cellular accumulation of cholesterol (Radhakrishnan et al., 2008). In the ER, excess cholesterol is esterified for storage as cholesteryl esters, further preventing the accumulation of excess cholesterol in the PM.

**Figure 9.**
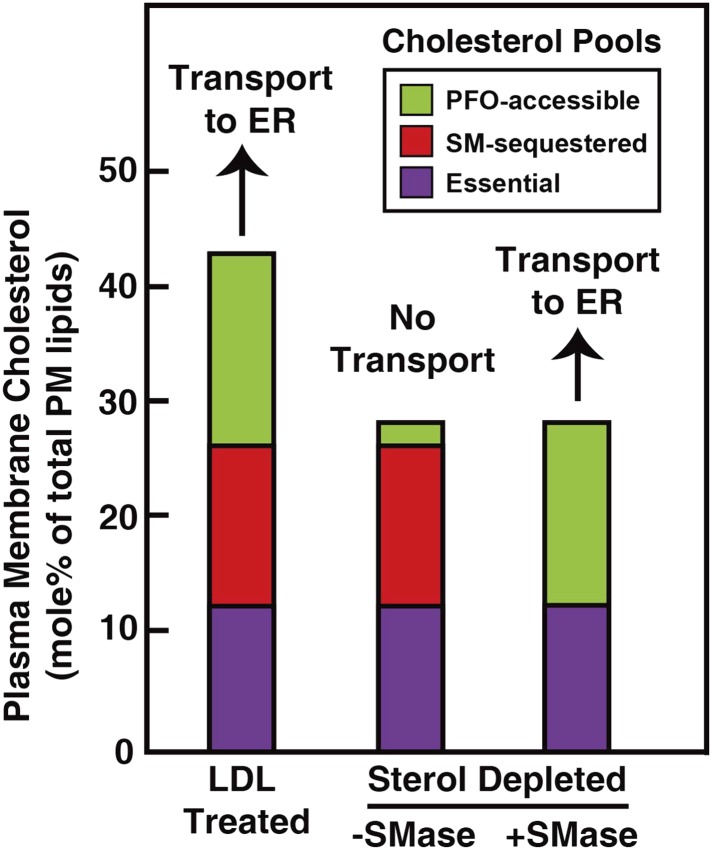
Schematic diagram illustrating the three pools of cholesterol in the PM of human fibroblasts under different conditions. **DOI:** http://dx.doi.org/10.7554/eLife.02882.013

Our analysis of the current results suggests that the PM contains three pools of cholesterol, as illustrated in Figure 9. One pool is accessible to ^125^I-PFO* binding (shown in green). In LDL-treated cells, the PFO-accessible pool accounts for 16% of PM lipids. The second pool, shown in red, is inaccessible to ^125^I-PFO, but becomes accessible when cells are treated with SMase. The size of this SM-sequestered pool, as determined from five independent experiments (Figures 3–6, 8), ranges from 10 to 23% of total PM lipids, with a mean of 15%. The third pool, shown in purple, is termed the ‘essential pool’. This pool was also determined from the aforementioned five experiments; it accounts for 12% of PM lipids and is not accessible to ^125^I-PFO*, even after SMase treatment. We call it ‘essential’ because reducing this pool with a high concentration of HPCD (>2%) causes cells to round up and dissociate from the petri dish, which indicates that this pool is essential for PM integrity. Although all of the results in the current study were done with human fibroblasts, we also found that the PM of CHO cells contains three pools of cholesterol (Figure 3—figure supplement 1). Whether these findings apply to other cell types grown under various conditions needs to be tested in future studies.

When cells are depleted of sterols by treatment with compactin in lipoprotein-deficient serum, the PFO-accessible pool becomes depleted (Figure 3). Even though the other two pools of cholesterol remain in compactin-treated cells, there is no transport to the ER and no cholesterol esterification. When sterol-depleted cells are treated with SMase, the SM-sequestered pool is released, and the liberated cholesterol becomes accessible for ^125^I-PFO binding (Figure 3A), restoring the PFO-accessible pool. Under these conditions even without the addition of LDL, a small amount of cholesterol is transported from PM to ER where it is esterified (Figure 6B).

The accessibility of only a portion of PM cholesterol to PFO is consistent with earlier work by Lange et al. (1980), who showed that only a portion of PM cholesterol is susceptible to the enzyme cholesterol oxidase. It seems likely that both PFO and cholesterol oxidase recognize the same pool of accessible cholesterol in the PM. While our work does not reveal the molecular basis of cholesterol accessibility, it is worth noting that studies on model lipid membranes have suggested a thermodynamic basis for this pool of accessible cholesterol (Radhakrishnan and McConnell, 2000; Lange et al., 2013). According to these studies, at low concentrations of cholesterol, most of the cholesterol is tied up in complexes with phospholipids and would not be accessible to soluble proteins such as PFO or cholesterol oxidase. At higher concentrations of cholesterol, the bilayer phospholipids become limiting, and a pool of cholesterol emerges that is not tied up in complexes with phospholipids. It is this ‘free’ cholesterol that is accessible to PFO (or cholesterol oxidase). The free cholesterol concentration is related to thermodynamic quantities termed chemical activity or fugacity (Radhakrishnan and McConnell, 2000).

The portion of PM cholesterol that is inaccessible to extracellularly added PFO could theoretically be sequestered either in the extracellular or in the cytoplasmic PM leaflets by membrane phospholipids. This PFO-inaccessible pool is subdivided into the SM-sequestered pool and the essential pool. Loss of SM upon SMase treatment increases the chemical activity of cholesterol in the outer leaflet of the PM and expands the pool of PFO-accessible cholesterol. Some of this accessible cholesterol could be transferred to the inner leaflet of the PM by cholesterol flip-flop, but there is no mechanism in the PM to convert this excess cholesterol into an inert form as ACAT does in the ER membrane. Instead, the excess PM cholesterol is transported to the ER membrane by vesicular or non-vesicular pathways to shut down SREBP processing and to be converted into cholesteryl esters by ACAT (Scheek et al., 1997; Abi-Mosleh et al., 2009). The physical nature of the SM-sequestered pool of cholesterol is not known, although a model of cholesterol-SM complexes is plausible based on studies in model membranes (Radhakrishnan and McConnell, 2005; Lönnfors et al., 2011; Lange et al., 2013). The idea of complexes of cholesterol and phospholipids is not new: more than 60 years ago, an X-ray diffraction study of nerve fibers suggested a 1:1 complex between cholesterol and phospholipid as the structural basis of the myelin sheath (Finean, 1953). Higher-order structures such as the proposed lipid raft domains rich in SM and cholesterol provide an additional mechanism for cholesterol sequestration.

The existence of an essential pool of PM cholesterol is inferred from the data of Figure 8 in which PM cholesterol was depleted by incubation of the cells with varying concentrations of HPCD. At the highest HPCD concentration tested (2%), the PFO-accessible pool was totally depleted, and the SM-sequestered pool was reduced by 80% (red curve in Figure 8). At this point, PM cholesterol was reduced from the control value of 45–22% of total PM lipids. Despite this severe reduction in PM cholesterol, the cells appeared normal by phase contrast light microscopy. When we increased the HPCD concentration above 2%, the morphology of the cells was drastically altered. The cells rounded up and many of them dissociated from the petri dish. Thus, the PM cholesterol that is retained at 2% HPCD is essential for normal PM morphology. Inasmuch as most of this residual cholesterol is neither PFO-accessible nor SM-sequestered, we term it the essential pool. The physical nature of this pool is unknown, and it may well represent multiple subpools of cholesterol in complex with PM phospholipids other than SM. The further delineation of the molecular nature of all three pools of PM cholesterol in fibroblasts and other cell types is a challenge for future membrane structural biology.

## Materials and methods

### Materials

We obtained [1-^14^C]oleic acid (55 mCi/mmol) from American Radiolabeled Chemicals, St. Louis, MO; paraformaldehyde from Electron Microscopy Sciences, Hatfield, PA; *S. aureus* SMase from Sigma, St. Louis, MO; and monoclonal anti-His antibody from GE Healthcare, Pittsburgh, PA. All other reagents (tissue culture supplies, 2-hydroxypropyl-β-cyclodextrin (HPCD), methyl-β-cyclodextrin (MCD), ^125^I-NaI, LDL, lipoprotein-deficient serum, and stock solutions of sodium mevalonate and compactin were obtained from sources or prepared as previously described (Das et al., 2013). A stock solution of cholesterol/MCD complexes was prepared at a final concentration of 2.5 mM and a cholesterol/MCD ratio of 1:10 (Brown et al., 2002).

### Buffers and culture media

Buffer A contains 25 mM Hepes-KOH (pH 7.4), 150 mM NaCl, and 0.2% (wt/vol) bovine serum albumin. Medium A is DMEM (with L-glutamine) containing 100 units/ml of penicillin, 100 μg/ml streptomycin sulfate, and 10% (vol/vol) FCS. Medium B is DMEM (with L-glutamine) containing 100 units/ml penicillin, 100 μg/ml streptomycin sulfate, and 1% (vol/vol) Insulin-Transferrin-Selenium. Medium C is DMEM (with L-glutamine) containing 100 units/ml penicillin, 100 μg/ml streptomycin sulfate, and 5% (vol/vol) newborn calf lipoprotein-deficient serum. Media D and E are identical to media C and B, respectively, except for the absence of L-glutamine in the DMEM in media D and E. Medium F is 1:1 mixture of Ham's F-12 medium and DMEM (with L-glutamine) containing 100 units/ml penicillin, 100 μg/ml streptomycin sulfate, and 5% (vol/vol) FCS. Medium G is 1:1 mixture of Ham's F-12 medium and DMEM (with L-glutamine) containing 100 units/ml penicillin, 100 μg/ml streptomycin sulfate and 5% (vol/vol) newborn calf lipoprotein-deficient serum. Medium H is DMEM (without L-glutamine) containing 100 units/ml penicillin and 100 μg/ml streptomycin sulfate.

### Cell culture

Stock cultures of human SV-589 fibroblasts (Yamamoto et al., 1984) were grown in monolayer at 37°C in a 5% CO_2_ incubator and maintained in medium A. Stock cultures of hamster CHO-K1 and CHO-7 (Metherall et al., 1989) were grown in monolayer culture at 37°C in a 8–9% CO_2_ incubator and maintained in medium F and G, respectively.

### Purification and iodination of His-tagged PFO and PFO^*^

PFO refers to the fully active cytolytic form of the toxin (Flanagan et al., 2009); PFO^*^ refers to a mutant PFO in which tyrosine-181 was changed to alanine, yielding a version that is not cytolytic at 4°C (Das et al., 2013). Both PFO (Sokolov and Radhakrishnan, 2010) and PFO^*^ (Das et al., 2013) contained His6 tag at the NH_2_-terminus. The proteins were overexpressed in *Escherichia coli* and purified as described in the indicated reference. PFO^*^ was radiolabeled with ^125^I as previously described (Das et al., 2013).

### Membrane purification

The procedure for purification of PMs from SV-589 cells was carried out by cell surface biotinylation followed by streptavidin affinity chromatography as previously described (Das et al., 2013). ER membranes from SV-589 cells were purified by differential gradient centrifugation as previously described (Radhakrishnan et al., 2008).

### ^125^I-PFO^*^ binding to surface of cultured cells

Prior to addition of ^125^I-PFO^*^, cells were washed as follows to remove surface-bound lipoproteins or HPCD: three rapid washes with buffer A at room temperature, followed by two 10-min washes with the ice-cold buffer A in a 4°C cold room. After these five washes, each 60-mm dish of cells was incubated at 4°C with 2 ml of buffer A containing ^125^I-PFO^*^ as described in Legends. After the indicated time, cell monolayers were washed rapidly three times with ice-cold PBS, dissolved with 1 ml of 0.1 N NaOH, and shaken on a rotary shaker for 15 min at room temperature. Aliquots (500 μl) of the dissolved cells were removed for scintillation counting in a gamma counter and for measurement of protein concentration (50 μl) (Lowry et al., 1951). The data are expressed as μg ^125^I- PFO^*^ bound per mg cell protein.

### PFO binding to purified membranes

Each 100-μl reaction mixture contained 0.5 μg of PFO and either purified ER membranes (12 μg protein) or purified PMs (90 μg protein). After incubation for 1 hr at 37°C, each mixture was combined with 5 × SDS loading buffer, incubated for 10 min at room temperature, and then subjected to 10% SDS-PAGE, followed by immunoblot analysis using an anti-His antibody (2.6 μg/ml).

### Lipid measurements

Cell lysates were divided in half, and the PMs from both halves were purified as described above. For one half, the content of unesterified cholesterol and choline-containing phospholipids was determined as previously described (Das et al., 2013). Total phospholipids were estimated by multiplying the measured choline content by 1.53 to account for the proportion of phospholipids that do not contain choline (Das et al., 2013). The second half was used to measure SM and ceramide content. The purified membranes were homogenized by sonication in 1.5 ml of 25 mM HEPES (pH 6.8) buffer. Immediately afterward, 20 μl of a Ceramide/Sphingolipid Internal Standard Mixture II (Avanti Polar Lipids, Alabaster, AL) was added, and the mixtures were vortexed and sonicated at 40°C for 10 min. Lipids from the homogenate were extracted with 2 ml of 85:15 (vol/vol) ethyl acetate:isopropanol. SM and ceramide levels were resolved and detected using high-performance liquid chromatography (HPLC) coupled to a triple quadrupole mass spectrometer (MS; ABSciex, Framingham, MA) through an electrospray ionization interface (Shaner et al., 2009; Hammad et al., 2010). The mole% of individual lipid classes is defined as the moles of the indicated lipid divided by the sum of the moles of cholesterol, phospholipids, and ceramide. The total moles of the four most abundant species of SM and of ceramide, as determined by MS analysis, are referred to as total SM and total ceramide, respectively (Table 1).

### Cholesterol esterification assay

The rate of incorporation of [^14^C]oleate into cholesteryl [^14^C]esters and [^14^C]triglycerides by monolayers of SV-589 cells was measured as previously described (Goldstein et al., 1983).
